# Socioeconomic deprivation and barriers to live-donor kidney transplantation: a qualitative study of deceased-donor kidney transplant recipients

**DOI:** 10.1136/bmjopen-2015-010605

**Published:** 2016-03-02

**Authors:** Phillippa K Bailey, Yoav Ben-Shlomo, Charles R V Tomson, Amanda Owen-Smith

**Affiliations:** 1 School of Social and Community Medicine, University of Bristol, Bristol, UK; 2 Department of Renal Medicine, Freeman Hospital, Newcastle upon Tyne Hospitals NHS Foundation Trust, Newcastle upon Tyne, UK

**Keywords:** NEPHROLOGY, SOCIAL MEDICINE, QUALITATIVE RESEARCH

## Abstract

**Objectives:**

Socioeconomically deprived individuals with renal disease are less likely to receive a live-donor kidney transplant than less-deprived individuals. This qualitative study aimed to identify reasons for the observed socioeconomic disparity in live-donor kidney transplantation.

**Design:**

A qualitative study using face-to-face in-depth semistructured interviews.

**Setting:**

A UK tertiary renal referral hospital and transplant centre.

**Participants:**

Purposive sampling was used to select deceased-donor transplant recipients from areas of high socioeconomic deprivation (SED) (19 participants), followed by a low SED comparison group (13 participants), aiming for maximum diversity in terms of age, gender, ethnicity, primary renal disease and previous renal replacement therapy.

**Methods:**

Participants were interviewed following their routine transplant clinic review. Interviews were digitally audio-recorded and transcribed verbatim. Transcripts were coded using NVivo software and analysed using the constant comparison method described in Grounded Theory.

**Results:**

Themes common and distinct to each socioeconomic group emerged. 6 themes appeared to distinguish between individuals from areas of high and low SED. 4 themes were distinct to participants from areas of high SED: (1) Passivity, (2) Disempowerment, (3) Lack of social support and (4) Short-term focus. 2 themes were distinct to the low SED group: (1) Financial concerns and (2) Location of donor.

**Conclusions:**

Several of the emerging themes from the high SED individuals relate to an individual's lack of confidence and skill in managing their health and healthcare; themes that are in keeping with low levels of patient activation. Inadequate empowerment of socioeconomically deprived individuals by healthcare practitioners was also described. Financial concerns did not emerge as a barrier from interviews with the high SED group. Interventions aiming to redress the observed socioeconomic inequity should be targeted at both patients and clinical teams to increase empowerment and ensure shared decision-making.

**Strengths and limitations of this study**

- In-depth qualitative interviewing is the ideal methodological approach for understanding in detail the knowledge, beliefs, and experiences of renal patients.
- Participants who had received a deceased-donor renal transplant were interviewed to allow a focus on barriers to live-donor kidney transplantation rather than transplantation in general.
- Saturation was assessed as having been achieved.
- An important limitation is that this was a UK study with few participants from ethnic groups other than white, thus findings may not be transferable to populations in other health systems or ethnic groups.
- An area-level measure of socioeconomic deprivation, rather than an individual-level measure, was used to sample participants.

## Introduction

Live-donor kidney transplantation offers the best treatment in terms of life-expectancy and quality of life^1–6^ for most people with end-stage renal disease (ESRD). The possible long-term risks of live-donor nephrectomy are small,^7–10^ with evidence of good medium-term outcomes. The quality of life of most living kidney donors is at least equal to the general population, and usually returns to predonation levels after donation.^11–14^

Within the UK, a country with universal healthcare that is free at the point of access, people from socioeconomically deprived populations are more likely to have Chronic Kidney Disease,^15^ ^16^ more likely to require dialysis,^17^ ^18^ but less likely to receive a live-donor kidney transplant (LDKT)^19^ compared with less-deprived populations. The same has been demonstrated in the Netherlands,^20^ the USA^21–23^ and Australia.^24^ In the UK the observed difference persists in cohorts from which those with malignancy and those dying within the first 3 years of starting dialysis have been excluded, suggesting that recipient fitness cannot fully explain the observed socioeconomic disparity.^19^ A USA Consensus Conference in 2014 on Best Practices in Live Kidney Donation concluded that there is a need to understand the mechanisms behind these observed disparities and identify targets for intervention.^25^

A recent thematic synthesis has collated qualitative work exploring renal patient attitudes towards live-donor kidney transplantation.^26^ Patients’ concerns include feelings of guilt and indebtedness to the donor, and concern about the effect of donation on the donor’s health, employment, and financial situation.^27^ ^28^ Many studies have focused on the attitudes of minority ethnic groups,^29–31^ but, while recognising the interaction between ethnicity and socioeconomic position, to our knowledge little work has focused on the attitudes and experiences of socioeconomically deprived individuals.

Gaps remain in our understanding of barriers to transplantation experienced by socioeconomically deprived individuals.^26^ This study was designed to address this; to gain an in-depth understanding of the transplant knowledge, beliefs, and decision-making experiences of renal patients from areas of high socioeconomic deprivation (SED). Ultimately we aimed to identify barriers to live-donor kidney transplantation that might explain the socioeconomic inequity currently observed and may be amenable to intervention.

## Methods

### Participant selection

Participants were eligible if they had been recipients of a deceased-donor renal-only transplant at the regional transplant centre Southmead Hospital, North Bristol NHS Trust, Bristol, UK between 1 August 2008 and 31 July 2013. Individuals had been unable to identify any living donors, and/or had recruited donors who had not completed clinical assessment, and/or had declined offers from potential living donors. These reasons were not mutually exclusive and could differ by potential donor for the same recipient. Deceased-donor transplant recipients were selected as participants for the following reasons. We aimed to identify barriers to live-donor kidney transplantation experienced by people who were eligible and medically suitable for transplantation, which includes transplant waiting list (‘wait-listed’) patients and recipients of transplants. However, we wanted to collect data from individuals who had definitely not received a LDKT, rather than wait-listed patients who may still go on to receive a LDKT. Finally, we wanted to try to focus on barriers to live-donor transplantation rather than to transplantation in general, and therefore interviewed recipients of deceased-donor transplants.

The Index of Multiple Deprivation (IMD) 2010 score was used as a measure of SED at the small area level, derived from postcode data. This measure is based on methodology developed at the University of Oxford Social Disadvantage Research Centre^32^ and is based on routine census data. Each country in the UK has individual components constituting an IMD score.^33^ There are seven domains of deprivation (Income, Employment, Health and Disability, Education Skills and Training, Barriers to Housing and Services, Living Environment Deprivation, and Crime) that determine the index score for an area in England, with higher scores indicating greater deprivation. Individuals are linked to specific Lower Layer Super Output Area IMD scores based on their postcode. Lower Layer Super Output Areas comprise a set of adjacent areas of consistent size with similar social characteristics, typically containing a population of around 1500.

IMD scores can be nationally divided into five equal-sized population quintiles according to the level of deprivation of the output area to which they belong, with the fifth decile representing the greatest deprivation. The hospital patient identification records which were used to sample participants do not record individual-level socioeconomic status data. Therefore, the IMD area-level measure of socioeconomic position was used as an ecological proxy for individual socioeconomic position. The socioeconomic disparity in LDKT uptake that this study is aiming to investigate was demonstrated using the Townsend Index,^19^ an area-level measure of deprivation.^34^ Individual socioeconomic variables (employment status, marital status and number of dependents) were collected during interviews and from the full detailed medical records after participants had consented to participation.

Purposive sampling was used to select interviewees from areas of high deprivation (IMD quintiles 4–5) but aiming for maximum diversity in terms of age, gender, ethnicity, primary renal disease,^35^ and previous renal replacement therapy (RRT). Participants were invited in stages. Sample size was determined by reaching theme saturation, when few or no new concepts were emerging.^36^ ^37^ All individuals invited were over 18 years of age with the capacity to give consent to participation.

When the lead researcher deemed that theme saturation had been reached purposive sampling was used to invite deceased-donor renal transplant recipients from areas of lower SED (IMD quintiles 1–3), to further investigate the themes arising, and gain insights through comparison. Sample size was again determined by reaching theme saturation.

### Data collection

Participants were invited to participate by post with a letter from the renal consultant responsible for their care. Non-responders were sent two reminder letters. Invitations were staggered. From the eligible study population, 61 individuals were invited to participate. In total, 32 (52%) agreed to participate. Reasons for non-participation were not explored. Between February 2014 and July 2015 a face-to-face semistructured interview was conducted with each participant at the hospital following the interviewee's routine transplant clinic appointment. Participants were interviewed after their clinic appointment for their convenience. In addition, the research team felt that the discussion of relationships with family members, particularly around the sensitive issue of living donation, might be more openly and comfortably discussed away from the home where family members may be present. The interviews were conducted by the first author PKB. PKB was working as a full-time doctoral researcher at the time of the study. She had previously worked as a doctor at the hospital, and had been involved in the ward based and outpatient care of six of the participants. PKB had received formal training in Qualitative Methods, Qualitative Appraisal and the use of NVivo for qualitative analysis, on taught courses at the University of Bristol and the University of Surrey. PKB had received informal guidance and training from AO-S and other medical sociologists and anthropologists at the School of Social and Community Medicine, University of Bristol. Participants were informed in the written patient information sheet, and verbally prior to the interview, that the research team wanted to understand why some people received a LDKT while others did not. In particular, participants were told that certain groups of people appeared to be less likely to receive LDKTs than others, and that the research team wanted to understand the reasons for this. Participants were not told that the researchers were interested in socioeconomic factors as the researchers did not want to bias responses to have this focus. Participants provided written consent. All participants were able to speak English fluently and therefore translators were not required. Six individuals requested that a family member be present for the interview. Interviews explored individuals’ knowledge of transplantation. Interviews investigated recipients’ beliefs about live-donor kidney transplantation and their experiences (if any) of trying to recruit living donors. Attitudes to directed and non-directed live-donor kidney transplantation were explored. Antibody incompatible transplantation is defined as transplantation across an incompatible ABO blood group barrier, or across a human leucocyte antigen (HLA) antibody barrier, with defined donor-specific antibody being present at the time of transplantation.^38^ Desensitisation techniques to allow antibody incompatible transplantation were only introduced in the study centre in 2010, so knowledge regarding incompatible transplant options was not explored in detail as these options would not have been available for all study participants. A semistructured topic guide was employed to ensure that the main questions were discussed with all respondents (see online supplementary material—topic guide). Interviews lasted between 18 and 72 min. Interviews were digitally audio-recorded, transcribed verbatim and anonymised.

10.1136/bmjopen-2015-010605.supp1 Supplementary data

### Data analysis

The research design and analysis were informed by Grounded Theory methodology:^39^ ^40^ data collection and analysis were conducted concurrently, employing an iterative and inductive approach. Recordings were listened to and transcripts read twice for familiarisation. NVivo qualitative software was used to aid analysis. Sections of text of the transcribed interviews were coded by assigning descriptive labels, and codes were grouped on the basis of shared properties to create concepts, then categories, then themes, using the constant comparison method described in Grounded Theory.^39^ ^40^ All transcripts were coded by the first author (PKB). A subset of the interview transcripts was independently inductively analysed by another experienced qualitative researcher (AO-S), and coding discrepancies discussed to maximise rigour and reliability. Themes were identified and analytic induction used to identify any patterns arising. Following theme saturation among participants from areas of higher SED, the same approach was taken to the analysis of interviews with participants from areas of lower deprivation, further investigating the themes that had been generated. Thus themes common and distinct to each group emerged. All illustrative quotations were approved by study participants prior to publication.

The Consolidated criteria for reporting qualitative studies (COREQ)^41^ was adhered to.

## Results

In total, 32 (52%) of 61 invited individuals agreed to participate (table 1). Invited individuals from areas of high SED were less likely to agree to participate (see online supplementary table S1).

10.1136/bmjopen-2015-010605.supp2 Supplementary table Participant and Non-participant characteristics

**Table 1:** Participant characteristics

	High deprivation group N=19	Low deprivation group N=13
Characteristics	Number (%)	Number (%)
Sex		
Female	10 (53)	7 (54)
Male	9 (47)	6 (46)
Age group (years)		
21–40	3 (16)	1 (8)
41–60	10 (53)	5 (38)
61–80	6 (32)	7 (54)
Ethnicity		
White	16 (84)	13 (100)
Other ethnic groups*	3 (16)	0
Marital status		
Single	4 (21)	0
Married/long-term partner	11 (58)	12 (92)
Other (divorced or widowed/bereaved)	4 (21)	1 (8)
Number of dependents		
0	14 (74)	10 (77)
1	2 (11)	1 (8)
2 or more	3 (16)	2 (15)
Renal replacement therapy prior to transplantation		
None (chronic kidney disease 4/5)	1 (5)	1 (8)
Peritoneal dialysis	4 (21)	6 (46)
Haemodialysis	12 (63)	4 (31)
Peritoneal and haemodialysis†	2 (11)	2 (15)
Primary renal disease group‡		
Glomerular disease	4 (21)	4 (31)
Tubulointerstitial disease	5 (26)	1 (8)
Systemic disease affecting the kidney	3 (16)	0
Familial/hereditary nephropathies	5 (26)	3 (23)
Miscellaneous renal disorders	2 (11)	5 (38)
Employment status§		
Full time/part time employment	7 (37)	5 (38)
Unemployed—seeking/not seeking employment	4 (21)	0
Retired and other (eg, student, homemaker)†	8 (42)	8 (62)
Index of Multiple Deprivation quintile		
5 (most deprived)	6 (32)	0
4	13 (68)	0
3	0	5 (38)
2	0	2 (15)
1 (least deprived)	0	6 (46)

*This includes Black/African/Caribbean/Black British, Asian/Asian British, Mixed/multiple ethnic groups and other ethnic groups.

†One individual had received a live-donor kidney transplant which had failed, as well as peritoneal and haemodialysis prior to receiving a deceased-donor kidney transplant.

‡European Renal Association—European Dialysis and Transplant Association (ERA-EDTA) Registry Primary Renal Disease coding system.

§Groups combined as one individual in one group.

Common themes arose describing barriers to live-donor kidney transplantation, emerging from interviews with both the high and low SED groups (see online supplementary figure S1). Six themes appeared to distinguish between high and low SED groups (see online supplementary figure S2).

10.1136/bmjopen-2015-010605.supp3 Supplementary figure 1

10.1136/bmjopen-2015-010605.supp4 Supplementary figure 2

Four themes emerged almost exclusively from interviews with participants from areas of high SED: (1) Passivity, (2) Disempowerment, (3) Lack of social support and (4) Short-term focus. Two themes emerged almost exclusively from interviews with the lower SED group: (1) Financial concerns and (2) Location of donor. ‘Transplant knowledge’ is reported in detail due to its important relationship with the other emergent themes. Illustrative quotations are provided in the text alongside each theme.

### Themes emergent from high SED group

#### Passivity

Individuals from areas of high deprivation reported adopting a passive role in their interactions with clinicians. Participants described deferring to the judgement of clinicians without question or challenge, with many participants assuming any judgement made by a clinician was in their interests:

I went into it a bit blind and I just went with the flow, what people were telling me to do. I didn't look it up anything, I didn't take charge of my—I didn't take charge of anything really. I let people do it for me because I was scared and I didn't really want to know any details. (Interviewee 16)

Clinicians were reported as having not discussed live-donor transplantation at all (see Disempowerment section), which participants assumed was because it was not an option for them:

I never, ever knew that I had to ask for a test or anything or that I could be tested. (Interviewee 9)

Other participants speculated that clinicians might have assumed they had discussed live-donor transplantation with their families and decided it was not an option for them, when this was not the case. For some individuals their passivity reflected their low expectations for their health and future:

Even when I was on dialysis I never thought I'd get [a transplant]. (Interviewee 13)

For some individuals their passivity reflected trust in their clinicians, but others described doubting or internally questioning a clinician's lack of discussion of live-donor transplantation, and other decisions, but still not seeking a second opinion. Individuals reported not seeking a second opinion because of a lack of confidence in their ability to do this, and a lack of awareness of how this could be achieved.

#### Disempowerment

Disempowerment emerged from participants describing situations in which they felt clinicians had failed to adequately empower them in discussions regarding their own care and discussions with potential donors:

It was just something I didn't want to have to ask anyone ‘Can I have one of your kidneys please?’ How do you even start to approach that subject? (Interviewee 17)

I never, ever knew that I had to ask for a test or anything or that I could be tested. (Interviewee 9)

Participants described clinician paternalism and a lack of shared decision-making.

Participants described the following, as a result of which they were disempowered:

(1) Failure on the clinician's part to raise live-donor kidney transplantation as an option, and failure to fully discuss it if the patient raised it:

But living donors, it wasn't discussed…I don't know. Maybe because, like I said, because I was in a- relatively well that (pause) they- they just thought what it- you're happy to wait for another donor and your family will know about it. I don't know. (Interviewee 16)

We never discussed having a living donor…I don't think there was ever a discuss—there was never a discussion of having a live donation. (Interviewee 14)

(2) Inadequate information on full transplant options—including pre-emptive transplantation, and the impact of one transplant on subsequent transplant options.

(3) Lack of information on living kidney donation:

It's a major thing ‘cos don't know nothing about the operation…nobody said nothing anyway so nobody said ‘oh there's a fifty percent risk that she's going to die’ or anything like that. Nobody mentioned any risks, they just give you a kidney and that's it and there we go, happily trotting off into the sunset together but it doesn't work like that, does it? (Interviewee 9)

One individual directly contrasted her experience of not being engaged in decision-making with the experience of another family member who she reported had ‘instigated’ their LDKT, reaching a joint agreement with their consultant.

One individual described in detail how he felt he had been disempowered by a single clinician. Although persistent in requesting being listed for transplantation he felt unable to challenge his clinician's judgement that he was not suitable for transplantation, not considering or feeling able to ask for a second opinion, or knowing how he could do this. He described how he had therefore remained on dialysis for years, and his wife, who had offered to donate a kidney, had left him, which he in part attributed to the burden his time on extended dialysis had placed on their life together:

[Clinician X] said no [to transplant] and that was no…how am I going to get round [clinician X], you know, so who else is there to ask? I kept asking and asking [clinician X] ‘Can I go on the transplant list?’ Every time I went, ‘Can I go on the transplant list?’ ‘No, no, ‘cos your heart can't take it’, you know, and then…[Clinician Y] took over…and my first words was to [clinician Y] ‘Can I go on the transplant list?’ and [clinician Y] looked through…said ‘Well the first thing we'll do, we'll get your heart tested.’ and it came back and [clinician Y] said ‘Yep, you can go on the transplant, it's passed.’ Whereas [clinician X] was telling me all this time, wasted…without any testing. [Clinician X] was playing God, you know…Although [clinician X] might have been brilliant at their job…to me [they] just seemed to decide who lived and who died. (Interviewee 9)

#### Lack of social support

The term ‘social support network’ describes the network through which an individual receives (and provides) emotional, physical, practical, informational, and relational assistance.^42^ Participants from areas of high SED often described lacking an adequate social support network, in which potential living donors would or could be found:

My mum's family was quite large but I never really had much to do with them so there was no one else. (Interviewee 5)

Many participants expressed the belief that none of their family or close friends would volunteer to donate, and therefore these individuals were not asked to consider donating. Many participants described having large families that were geographically proximate, but perceived a lack of social support from them:

Even though the relations all come from [town] and are [geographically] close. We're…not really close [socially] enough to anybody that can. (Interviewee 1)

Other individuals reported that they had small circles of social support; having small families or few close friends:

I mean I haven't got a massive circle of friends anyway and I know the ones that I have got, a few of them have got their own health issues anyway so… (Interviewee 23)

Several individuals described a lack of received social support when this had been sought, giving examples of approaching people they had initially felt were in their social support network, but who had then declined to donate. Some individuals described instances in which relationships with potential donors had then broken down (examples included marriages dissolving, and all communication between siblings ending):

Yeah. Yeah, before she left (pause) when everything was happy and happy sort of thing, you know, I think it was- she was going to give a kidney to somebody else and somebody else was going to give a kidney to somebody and somebody was going to give a kidney to me- like a triangle-…She was willing to do that- It didn't happen, um, (pause) ‘cos she left’. (Interviewee 9)

Relationship breakdown was often attributed to the impact of chronic renal disease (especially familial disease and haemodialysis) on the recipient and donor's shared quality of life.

Participants primarily described a lack of social support specifically with reference to the issue of living kidney donation, but many described how this represented a general lack of social support, sharing other examples not related to donation.

In contrast, individuals from areas of low SED described significant social support, from family, friends, social groups, and faith groups. In these instances often a number of offers of donation had been received, even if these did not proceed. These potential donor offers had arisen from potential donors being asked directly by the transplant candidate or by other social contacts on the candidate's behalf, while others had volunteered without being asked.

#### Short-term focus

Individuals, almost exclusively from areas of higher deprivation, described making transplant decisions without a long-term perspective, instead focusing on their immediate situation:

I knew you could have trans, like obviously talking to other patients, but once again I wasn't really thinking about- I just thought of what was going to go on now…I'm the sort of person that doesn't think five years ahead. I don't even try and think a year ahead. I think within the next couple of months, whatever. That's how I've really had to have been through dialysis now…my whole life has been basically not thinking too far ahead. (Interviewee 11)

This included not believing one would ever reach a point of needing a transplant and therefore not planning for one:

It was suggested that I go on the transplant list and I kind of thought well, okay, that's what you do…and it was just a bit, um, unreal and I didn't think it would happen and I didn't need one…I mean (pause) I think coming here (pause) I didn't even think beyond…- (Interviewee 16)

I always knew inevitably I would need a transplant but I kind of thought it would never happen. You don't- they're probably just telling me that but inevitably it did happen. I didn't really think about it. I don't think about anything until it's right just about to happen…I just switch off, honestly…Until the day comes I sort of deal with it then. (Interviewee 22)

Participants also described wanting ‘any transplant’ in order to stop dialysis, rather than the transplant which may be associated with the best or longest survival:

There was no decision, it was just grab whatever you can, I'll take that. Give me one out of a tramp in the gutter, I'll take it, whatever. Made no difference. To get off that dialysis, you know, was everything. (Interviewee 9)

As participants described being focused on the immediate and short-term situation, they also described that their attitudes towards and the decisions they had come to regarding transplantation changed over time as their health, social situations, and those of possible donors, changed. For example, many participants described initially not considering LDKT as an option while they felt well on dialysis, as it did not feel ‘immediately necessary’. However as they became more unwell on dialysis, many then considered a LDKT as a way of ending their current suffering.

### Themes emergent from low SED group

#### Financial concerns

Financial concerns were almost exclusively mentioned by people from areas of *lower* SED. Individuals were concerned by a donor's potential loss of earnings, especially in the context of self-employment:

I had a mate in [country] who said ‘I'll give you one’…but his problem would have been financial. He's got a no contract job, he lives from hand to mouth, I know that they can help after the operation if you can't work but just taking the days off for the tests would have, you know, put him under the—so I was very dubious about asking him cos officially I'm not allowed to give him any money but if I had some I'd have to because he's living on such narrow margins that that, I don't know, probably half a dozen days he'd need off for tests and, you know, that would have been- It could be the be all and end all for somebody who was already, like a lot of people are, you know, just breaking even. (Interviewee 6)

The financial impact if the donor and recipient were from the same household was raised as a particular concern:

I think specially, or with my husband obviously he's the breadwinner and he's a [job requiring a high level of physical fitness] and so there's things that I think—I can't compromise his health (pause) just in case ‘cos you just don't know. (Interviewee 26)

Many participants reported that they were not aware of any financial help for donors undergoing assessment. Other interviewees mentioned that reimbursement was inadequate as donors still experienced being ‘out of pocket’. Several participants had considered personally reimbursing a donor. The socioeconomic position of potential donors often appeared to differ from that of the recipient: one low deprivation group individual described a friend who had offered to donate to him as living ‘hand to mouth’, while a higher deprivation group individual described having a sibling who had ‘an international, high flying job’.

#### Location of donor

Potential donors living abroad or far away within the UK was described as a barrier to donation, only by individuals from the less-deprived areas:

It was [surgeon] I think I spoke to and he said about, you know, ‘Do you have anyone?’ and I said well, I explained as I said to you that my son lived in [Non-UK country] which was going to be very difficult…and he said ‘Oh fair enough’ so that was it really. We didn't get into a big conversation about it, it was just with him living that far it, you know, probably would involve several trips over which would be quite difficult. (Interviewee 8)

Participants assumed the donor's assessment would be carried out in the transplant centre at which the intended transplant recipient received their care, and the cost and the impact on employment and caring responsibilities were perceived by the interviewees as barriers to these individuals donating:

He was working in [UK country] so he would have had to have a day off and come up to here and we didn't talk about it but I was thinking he's not going to be able to do it, it's going to be too much, you know. (Interviewee 6)

And my brother lives in [Non-UK country] and he was working then…and he wouldn't have, he wouldn't have, he wouldn't if he had been able to, he would not have come over here for all that stuff and illness and, he wouldn't have done, too busy. (Interviewee 13)

#### Transplant knowledge

Evidence of limited transplant knowledge emerged from interviews with participants from all levels of deprivation. Most participants reported that there were benefits to having a LDKT, in terms of immediacy and quality of graft function, and graft ‘life-expectancy’:

I would have preferred a living one because you know you sort of think you've got a better chance… (Interviewee 5)

[I'd prefer] the living one, and I- the reason why is I assume, although I've been told the difference isn't that much, that they're going to live longer, it's going to be more, you know, it hasn't had a period of not being properly active, you know, not- That's my assumption- but I don't think there's (pause) the life of the kidney is only dependent on the health of the kidney. (Interviewee 16)

However, the benefits of a LDKT over a deceased-donor transplant were perceived as marginal and a couple of participants reported that there were no advantages to a LDKT.

I thought to get a kidney might prolong my life either way, a living donor or a deceased. That's what I think really. (Interviewee 7)

Most participants reported knowing that the risks of living donation were small, but reported a lack of information on the process of living donor evaluation:

People don't think they can do it, you know. They think, well I don't know- ‘How do you do it? How do you offer somebody a kidney, you know, how do you go about it?’ I don't really think that there's enough literature out there to say to people ‘It's okay’, you know, ‘you can actually do this’. (Interviewee 12)

Participants described how without this information they were unable to inform their potential donors of the evaluation they could expect to undergo. Many participants held beliefs about an upper age limit for living donation that may not have been valid:

If the discussion happened ten years ago my mother would have been 83. Too old to be a kidney donor. At 93 she is looking forward to Christmas. (Interviewee 18)

Gaps in knowledge regarding location of donor evaluation and reimbursement policies were also apparent.

## Discussion

This study identified patient-perceived barriers to live-donor kidney transplantation. The majority of themes were common to all interviewees, regardless of their level of SED. This finding suggests that there are universal barriers to live-donor kidney transplantation, affecting all potential recipients, albeit possibly to different degrees. Most of these themes have been previously well described.^26^ The major contribution of this study is the identification and description of those themes that appear to be distinct to high or low deprivation groups, which thus might explain the observed socioeconomic inequity, and provide targets for intervention.

### Financial barriers

Financial concerns regarding live-donor kidney transplantation have been well reported.^29^ ^43^ ^44^ Interestingly, in our study financial concerns were expressed almost exclusively by the less deprived. Differing healthcare funding models mean that the financial impact of a LDKT on donors and recipients will differ between countries, thus potentially limiting the transferability of this finding. However, reasons for this finding can be postulated. First, as stated above, several participants suggested that the socioeconomic position of their potential donors differed from their own socioeconomic position. If the potential donors of deprived intended recipients are not themselves deprived, then in some cases this might explain why financial barriers are not experienced or reported. Second, individuals and their potential donors in the UK who are unemployed and/or in receipt of welfare benefits may be less impacted financially by LDKT evaluation and surgery, as ‘income’ is not affected by the transplant evaluation process. In addition, donor–recipient pairs sharing one economic household may gain significant financial benefit from returning a chronically ill recipient to work, as suggested by previous work,^45^ and the impact of this might be greatest for those with low incomes from areas of high deprivation. Given this finding, efforts to reduce financial barriers to live-donor kidney transplantation,^25^ ^46^ although valuable, may not reduce the observed socioeconomic disparity in the UK. As previously noted, SED describes ‘a deprivation of basic capabilities due to a lack of freedom, rather than merely low income’.^47^

### SED and the interplay of themes

Limited transplant knowledge emerged as a barrier to live-donor kidney transplantation for all,^48^ and therefore alone it does not appear to explain the observed socioeconomic disparity. Rather, the findings of ‘passivity’ and ‘disempowerment’ suggest that the confidence an individual has in this knowledge and in applying this knowledge may differ with SED, including the confidence to question clinicians and engage in shared decision-making. These findings appear to support the results of a recent cross-sectional survey from the USA which found that how an individual *perceived* their transplant knowledge, and their confidence in this knowledge, was more important than their *actual* knowledge in pursuing a LDKT.^49^

‘Patient activation’ is a behavioural concept that incorporates the emergent themes of passivity, disempowerment and limited knowledge. It is defined as ‘an individual's knowledge, skill, and confidence for managing their health and healthcare’.^50^ ^51^ Participants from areas of high SED suggested lower levels of patient activation and this finding is in keeping with previous research.^52^ ^53^ Patient activation is not a fixed behaviour resultant from patient-related factors only. It is a dynamic continuum, and clinical teams have the ability, and arguably the responsibility, to improve an individual's activation. It emerged from our study that some patients did not feel adequately empowered by their clinicians, suggesting that there is room for improvement.

### Future research and intervention

How much the emergent themes explain the socioeconomic variation in live-donor kidney transplantation needs further investigation with qualitative and quantitative methodologies. Interviews with LDKT recipients from socioeconomically deprived areas may give us insight into whether the same barriers to transplantation were encountered, and how they were overcome. Likewise given the many universal themes, it would be interesting to explore whether patients’ strategies to cope with the same barriers differ according to their level of SED.

Specific gaps in knowledge were identified that might benefit from targeted education of patients and clinicians. Several studies have found that the addition of home-based education to standard clinic information, increases patient and potential donor knowledge about, and willingness to consider, LDKTs.^54–56^ Given the apparent focus of deprived patients on the immediate when making decisions regarding transplantation, education and discussion should be repeated at multiple time points throughout the disease progression and transplant evaluation process.^57^

As already stated however, education alone may be insufficient to change the socioeconomic gradient in live-donor kidney transplantation. In addition to information and education, individuals require confidence and facilitated self-efficacy. Psychosocial empowerment interventions and strategies to tailor support to, or increase, an individual's level of patient activation^51^ ^58^ ^59^ should be trialled and may be effective at redressing the socioeconomic disparity in transplantation. Interventions could include tailored coaching,^58^ building question formulation skills with patients prior to their clinical interactions,^59^ and lay peer support from an individual who shares socioeconomic status, ethnicity and clinical experiences with the renal patient.^60^ Patient empowerment also requires commitment from clinicians to support shared decision-making. Interventions to improve the practice of healthcare teams in empowering and engaging patients have been trialled in the UK^61^ ^62^ and could be applied to the field of renal transplantation. Indeed, transplant shared decision-making aids exist^63^ but their use is not widespread, and challenges regarding their application must be overcome.^64^ As well as empowering patients themselves, additional approaches include the use of recipient advocates to support individuals lacking self-efficacy.^65^

### Limitations

There are some limitations: (1) This was a single-centre study from a UK transplant centre with few study participants from ethnic groups other than ‘white’ (n=3). Therefore, findings might not be transferable to populations in other health systems or ethnic groups. (2) The area-level IMD was used to measure SED and sample participants. The IMD may not reflect a specific individual's socioeconomic position. However individual measures of SED do not account for the other contextual effects that poverty and the environment impart on an individual that may be important here. In addition, individual measures of SED were recorded after participation which supported the IMD score classifications (ie, 21% of the high deprivation group were unemployed vs 0% in the low deprivation group; 42% of the high deprivation group lived as single as compared with 8% of the low deprivation group); (3) 48% of those invited to participate declined, with individuals from areas of high SED much more likely to decline. Reasons for non-participation were not explored, however, this finding may itself reflect the low levels of engagement of deprived individuals in healthcare reported in our study. In addition, the sampling strategy employed resulted in larger numbers of individuals from areas of high SED being invited to participate, until a sample size resulting in theme saturation was achieved; (4) Interviews were carried out by a clinician (PKB), previously known to six of the participants as a specialty trainee (registrar) who had been involved in their ward-based and outpatient care. Although participants spoke freely we are unable to determine if this altered responses; (5) Participants had all received a deceased-donor kidney transplant that may have altered their attitudes to live-donor kidney transplantation. However, participants discussed positive and negative experiences of deceased-donor transplantation, and the findings of this study are very similar to those of a study from the USA which studied participants with ESRD who had not received a transplant;^66^ (6) Finally, the two study groups were interviewed sequentially. Although every attempt was made to retain a ‘clean sheet’ inductive approach to analysis the coders will have been sensitised to themes arising in earlier interviews.

### Summary

The primary concern of this research team is to ensure individuals have equal access to the best RRT regardless of socioeconomic status. This study contributes new knowledge to our understanding of the reasons for the observed socioeconomic inequity in live-donor kidney transplantation, highlighting barriers within the intended recipients themselves and within the healthcare service. Targeted interventions should ensure the likelihood of receiving a LDKT is not determined by socioeconomic position.
