# Supplementary material for: Socioeconomic deprivation and barriers to live-donor kidney transplantation: a qualitative study of deceased-donor kidney transplant recipients
Source: BMJ Open. 2016 Mar 2;6(3):e010605. doi: 10.1136/bmjopen-2015-010605 (PMC4785291; doi:10.1136/bmjopen-2015-010605)
Supplement: Supplementary data [file bmjopen-2015-010605supp.pdf]

Topic Guide

Regarding initial experiences
Could you tell me a little bit about when you were first diagnosed with kidney problems?
Encourage through story – impact of renal disease on life, dialysis choice, impact of dialysis etc

Regarding information sources and understanding live donor kidney transplantation.
Can you tell me a bit about how you came to know a transplant might be an option for you?
Could you tell me about the information you had about transplants?
Explore: Sources of information; timing of information; preferred type and source; suitability of information; any missing information desired.

Regarding attitudes to live donor kidney transplantation
Can you tell me what you *think* about transplantation in general?
What's your personal view on live-donor kidney transplantation? Explore willingness to receive a live-donor kidney transplant (LDKT).
How do you think you came to hold that view?
Explore: Experiences impacting on view; Sources of information; Time to develop view and has it changed over time;
If you ever needed another transplant in the future, what would you think about a LDKT?
If led by interviewee, explore attitudes to directed and non-directed (unspecified/altruistic).

Regarding understanding/knowledge of transplantation and living donation
Can you tell me a bit about what you *know* about live donor kidney transplantation?
What do you understand the differences to be between deceased-donor and live-donor transplantation? Can you think of any advantages or disadvantages of one over the other?
Explore: What do you know about transplantation compared to dialysis? If you had a live donor and a planned date for the LDKT, but you were called and offered a deceased-donor kidney, can you tell me how you'd make a decision about which one to accept?
If mentioned by interviewee, explore knowledge of directed and non-directed (unspecified/altruistic).

What do you think about living *donation*?
Prompts: Does anything concern you about living kidney donation? Do you think there are any advantages/disadvantages to live donation for the donor?

Regarding considering potential donors
Can you tell me about any conversations you had with friends or family about live kidney donation?
Can you tell a bit about how these conversations came about?
Did you make a decision about LDKTs overall or did you consider each possible donor differently?

Prompts: Did you ask anyone if they'd consider donating a kidney, or think about asking anyone?
Why/why not? How did you decide who to ask? Any specific reasons why you did or didn't ask people?
Explore reasons (health, age, location, financial, lifestyle, employment etc)

Did anyone offer to donate a kidney to you? Could you tell me a bit about that?

Regarding considering potential donors
If it's okay with you, it'd be really useful to talk through your family and close friends in a bit more detail.
Explore why participants did or didn't consider them as possible living donors.
Suggest using checklist to make sure no-one is forgotten:
- Spouse/Partner, Parents (incl. step), Siblings (incl. step), Cousins, Children, Aunts/Uncles, Nieces/Nephews, Friends

Regarding barriers and facilitators
How did you find the process of thinking about LDKTs? Did anything make the process easier/more difficult?
If interviewee wanted LDKT, were there any things you think stopped you from having a LDKT?

Explore: Personal barriers (including caring responsibilities, financial, employment), family barriers, health system level barriers. If financial barriers raised, explore awareness of reimbursement.

Non-directed unspecified 'altruistic' donation
Have you heard about something called 'altruistic / Good Samaritan / non-directed' kidney donation? Explore knowledge/understanding.

Inform or clarify – 'this is a situation in which a stranger offers to donate a kidney whilst they're still alive, to someone they don't know, someone on the waiting list.'

What do you think about this? Explore attitudes.
Were you offered an 'altruistic' kidney transplant? What were your thoughts about this/what do you think your thoughts might be to this if you were offered this in the future? How did you/do you think you would feel?

Forced choice – hypothetical scenario 'If you were offered both an 'altruistic' live-donor transplant as well as a deceased-donor transplant, which would you pick? Would you have a preference for one over the other?
Can you talk me through your thinking / How you came to that decision? Explore the reasoning in detail.

e.g. Say you're told an altruistic kidney donor has been matched to you, and you're being offered their kidney, and the operation is due to go ahead but the day before the planned transplant you're offered a deceased donor kidney transplant – which would you pick?

Regarding future interventions
Is there anything you think might make the process easier? Anything that might help people to have living kidney transplants?
Explore: Use of recipient advocates; More/different information; Home education; 'One-stop (faster) donor work-up'; Financial support.
