# Supplementary material for: Socioeconomic deprivation and barriers to live-donor kidney transplantation: a qualitative study of deceased-donor kidney transplant recipients
Source: BMJ Open. 2016 Mar 2;6(3):e010605. doi: 10.1136/bmjopen-2015-010605 (PMC4785291; doi:10.1136/bmjopen-2015-010605)
Supplement: Supplementary table — Participant and Non-participant characteristics [file bmjopen-2015-010605supp_table.pdf]

Supplementary File – Table: Participant and Non-participant characteristics

Characteristics	Number invited	Participants (% of total invited)	Non-participants (% of total invited)
**Sex**			
Female	27	17 (63)	10 (37)
Male	34	15 (44)	19 (56)
**Age group (years)**			
21-40	11	4 (36)	7 (64)
41-60	30	15 (50)	15 (50)
61-80	20	13 (65)	7 (35)
**Ethnicity**			
White	49	29 (59)	20 (41)
Non-white	12	3 (25)	9 (75)
**Primary renal disease group**			
Glomerular disease	18	8 (44)	10 (56)
Tubulointerstitial disease	13	6 (46)	7 (54)
Systemic disease affecting the kidney	6	3 (50)	3 (50)
Familial/hereditary nephropathies	12	8 (67)	4 (33)
Miscellaneous renal disorders	12	7 (58)	5 (42)
**Index of Multiple Deprivation Quintile**			
5 (most deprived)	21	6 (29)	15 (71)
4	21	13 (62)	8 (38)
3	6	5 (83)	1 (17)
2	5	2 (40)	3 (60)
1 (least deprived)	8	6 (75)	2 (25)
