# Supplementary material for: Socioeconomic deprivation and barriers to live-donor kidney transplantation: a qualitative study of deceased-donor kidney transplant recipients
Source: BMJ Open. 2016 Mar 2;6(3):e010605. doi: 10.1136/bmjopen-2015-010605 (PMC4785291; doi:10.1136/bmjopen-2015-010605)
Supplement: Supplementary figure 1 [file bmjopen-2015-010605supp_figure1.pdf]

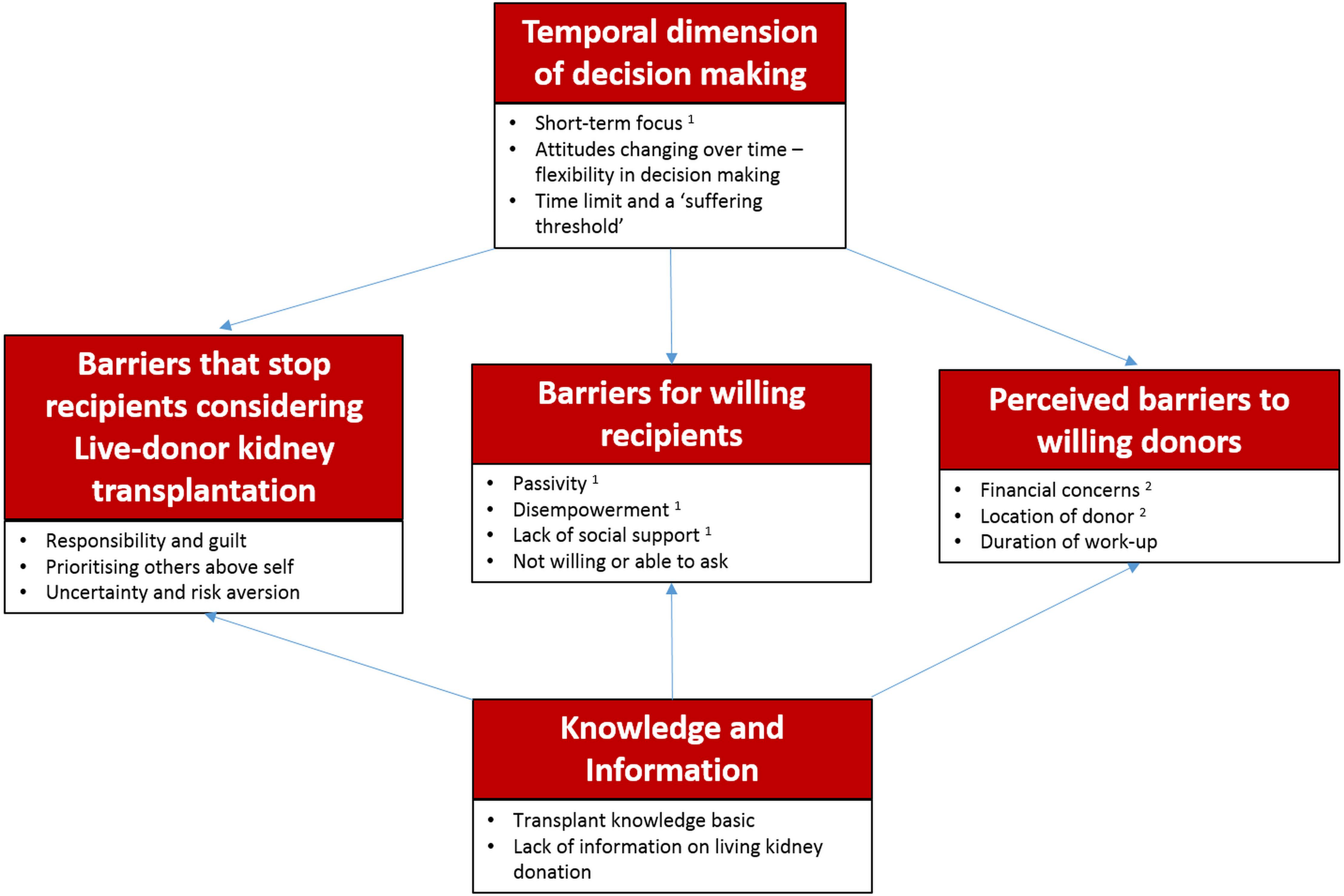

Figure 1: Thematic schema of barriers to live-donor kidney transplantation – demonstrating each step towards live-donor kidney transplantation

[1] Theme identified almost exclusively in interviews with individuals from areas of high socioeconomic deprivation

[2] Theme identified almost exclusively in interviews with individuals from areas of low socioeconomic deprivation.
