# Supplementary figures and images for: Socioeconomic deprivation and barriers to live-donor kidney transplantation: a qualitative study of deceased-donor kidney transplant recipients
Source: BMJ Open. 2016 Mar 2;6(3):e010605. doi: 10.1136/bmjopen-2015-010605 (PMC4785291; doi:10.1136/bmjopen-2015-010605)

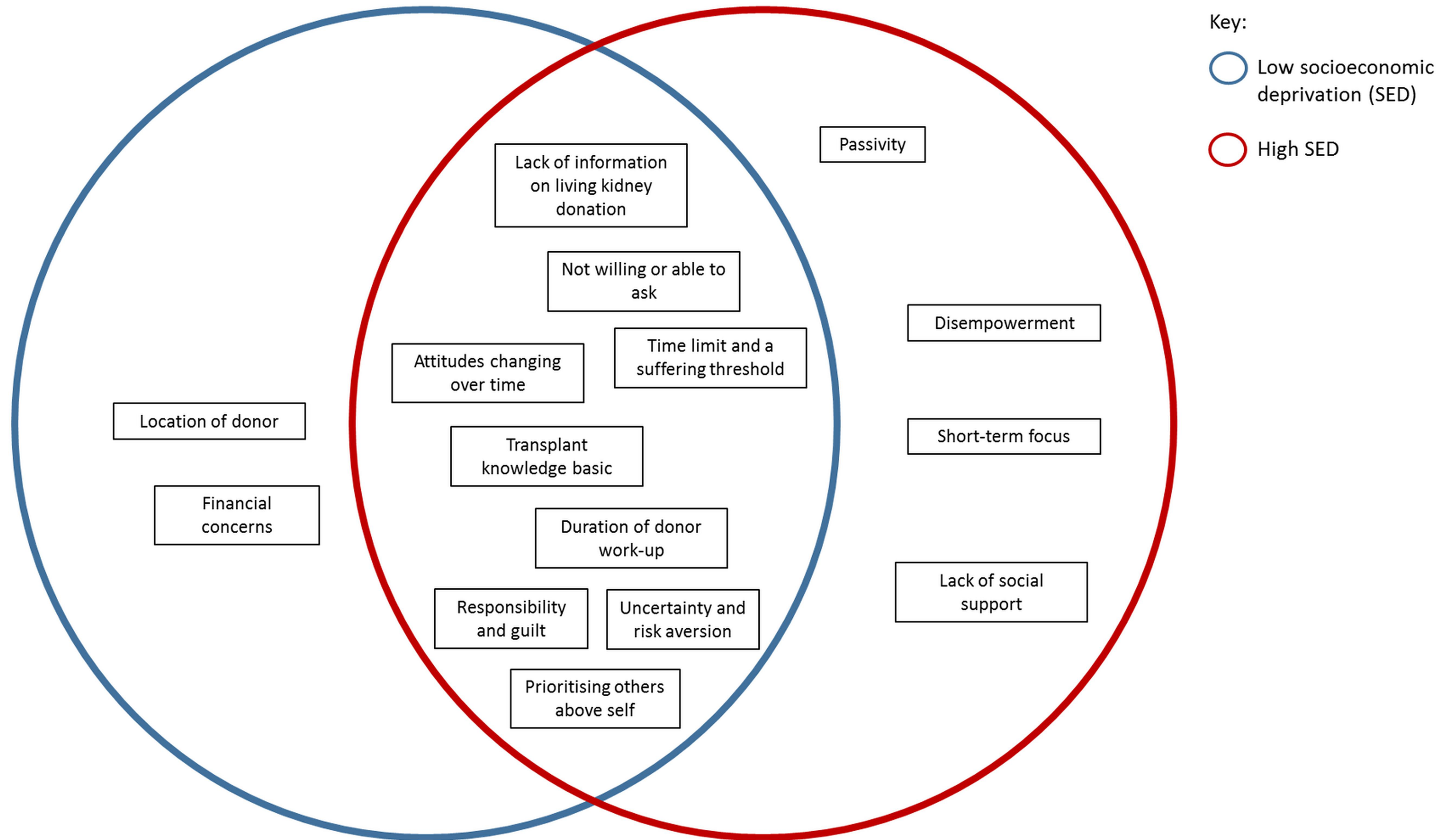

**Figure 2: Themes by level of socioeconomic deprivation**

Supplement: Supplementary figure 2 [file bmjopen-2015-010605supp_figure2.pdf]
